# New Regulations Respecting Teachers of Anatomy, &c.

**Published:** 1837-01

**Authors:** Edmund Belfour

**Affiliations:** Sec.


### NEW REGULATIONS RESPECTING TEACHERS OF ANATOMY, &c.

*Royal College of Surgeons.* The Council of the College, at an extraordinary meeting, on the 1st instant, established the following Ordinance, relating to the recognition of the certificates of teachers of anatomy and surgery in England and Wales:—

That in future no person be recognized by this College as a teacher of anatomy, physiology, and pathology, or in surgery, in England and Wales, until he shall have undergone an examination before the Council of the College on two separate days. The first examination to be in anatomy and physiology; the second in pathology, and on the principles and practice of surgery.

That no fee be demanded for these examinations; and that the recognition of the College be conveyed in the usual form of a letter from the Secretary.

By order, EDMUND BELFOUR, Sec.

*November* 18, 1836.

